# Stem Cells Spirited Away by Alcohol-Induced DNA Damage

**DOI:** 10.1097/HS9.0000000000000036

**Published:** 2018-03-30

**Authors:** Michael D. Milsom

**Affiliations:** 1 Division of Experimental Hematology, German Cancer Research Center (DKFZ), Heidelberg Germany; 2 Heidelberg Institute for Stem Cell Technology and Experimental Medicine (HI-STEM), Heidelberg, Germany.

The gradual acquisition of mutations in the genome of somatic cells is a hallmark of aging and leads to the evolution of a range of age-associated diseases, such as cancer. The accrual of such mutations within adult stem cells is thought to be of particular biological relevance, because these cells are long lived and can therefore accumulate a significant mutation burden during the lifetime of an organism, which is then propagated in all daughter cells. Somatic stem cells, or their immediate progeny, are also a likely cell of origin for malignant transformation. Although the DNA damage response of various tissue-specific stem cell types has been extensively explored using agonists such as ionizing radiation or chemotherapeutic agents, it is unclear what the major source of DNA damage is under physiologic conditions and how this is resolved into mutations within stem cells. A recent manuscript published in Nature sheds new light on this issue using the hematopoietic system as a model tissue to explore how by-products of the metabolism of alcohol can act as a mutagen within hematopoietic stem cells (HSCs), leading to their functional attrition and impaired hematopoiesis.^1^

Excessive use of alcohol has been identified as a causal factor in a wide range of diseases, including several different forms of cancer. Acetaldehyde is thought to be an important toxic metabolite of ethanol that can form adducts on DNA, and is normally detoxified by protective enzymes such as aldehyde dehydrogenase 2 (ALDH2). In previously published work, the group of K.J. Patel had uncovered the link between acetaldehyde and the Fanconi anemia (FA) DNA repair pathway, which is involved in resolving DNA interstrand crosslinks.^2,3^ Loss of function of the FA pathway in humans results in bone marrow failure (BMF) and cancer predisposition. Knockout mouse models of FA demonstrate analogous DNA repair defects to those seen in FA patient cells, yet never spontaneously develop BMF. The Patel group hypothesized that alcohol-mediated production of acetaldehyde could produce DNA damage intermediates that are normally resolved by the FA pathway. Using an elegant genetic approach, they could validate this hypothesis by showing that combinatorial loss of function of the FA repair pathway along with inactivation of *Aldh*2, led to elevated DNA damage within murine hematopoietic cells, followed by leukemogenesis and bone marrow failure. Importantly, these phenotypes were further exacerbated by the exposure of mice to alcohol. The clinical relevance of these findings was later underscored by the observation that FA patients who additionally harbored a common dominant negative polymorphism in the *ALDH2* gene, demonstrated an earlier onset of bone marrow failure compared to FA patients with normal ALDH2 function.^4^

In their most recent manuscript, the same group sought to further strengthen the mechanistic link between alcohol exposure and genotoxic damage, by focusing on a detailed characterization of the mutational burden in HSCs.^1^ Initial experiments focused on the evaluation of clastogenic damage in mouse blood cells. Assessment of the frequency of micronuclei present in normochromic erythrocytes (NCEs) was used as an assay to determine the degree of chromosome breaks within hematopoietic cells. Mice with individual loss of function of either *Aldh2* (*Aldh2*^−/−^) or the FA pathway (*Fancd2*^−/−^), both demonstrated elevated levels of NCEs with micronuclei compared with wild type control mice. However, combinatorial *Fancd2*^−/−^*Aldh2*^−/−^ mice had dramatically increased micronuclei burden in NCEs, illustrating the partial redundancy in these detoxification pathways. In line with this observation, M-FISH analysis revealed much higher frequencies of chromosomal abnormalities in bone marrow cells from double knockout mice compared with either single knockout alone. Importantly, alcohol exposure dramatically increased clastogenic damage in hematopoietic cells of *Fancd2*^−/−^*Aldh2*^−/−^ mice and was accompanied by a rapid collapse in the hematopoietic system leading to death from BMF.

**Figure 1:**
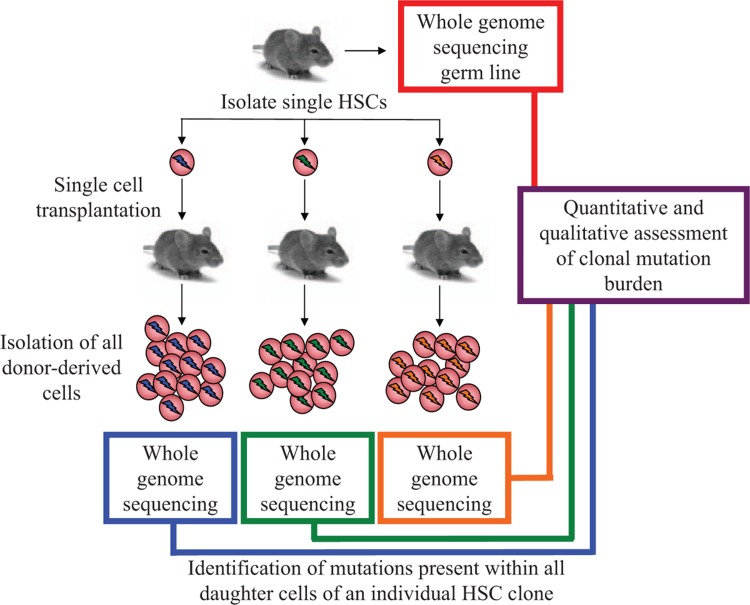
**Schematic representation of single-cell transplantation approach used to define mutation burden of functional HSCs at the clonal level.** Common mutations inherited from the original transplanted HSC are depicted as blue, green, or red mark.

In order to try and correlate whether the coordinate action of these detoxification pathways was important for maintaining the functional and genomic integrity of HSCs, the authors next went on to conduct a series of ambitious and technically challenging experiments to enumerate the mutation burden in individual HSC clones (Fig. 1). Individual HSCs were isolated from *Aldh2*^−/−^, *Fancd2*^−/−^, or *Aldh2*^−/−^*Fancd2*^−/−^ mice by flow sorting, following which irradiated recipient mice were transplanted with these single cells. At 4 months post-transplantation, all the mature blood cell progeny of the transplanted HSC were harvested and subject to whole genome sequencing. By comparing the sequencing data with the germline sequence obtained from a tail clipping of the original donor mouse, it was then possible to identify the somatic mutations that were present in all donor progeny cells and therefore must have been present in the transplanted HSC clone. The engraftment rate of the transplanted *Aldh2*^−/−^*Fancd2*^−/−^ HSCs was very low compared with control or single knock out HSCs, demonstrating that the HSCs were indeed functionally compromised. Comparative analysis of the mutation signature in HSCs showed a modest increase in point mutations in the double knockout cells, but a more profound increase in deletions and rearrangements. This indicates that endogenous acetaldehyde damage induces DNA double-strand breaks within HSCs in vivo, which are then resolved by error-prone end joining repair. Since p53 activation has been previously proposed to be a downstream consequence of loss of function of the FA pathway, which then mediates HSC depletion and BMF,^5^ the authors next generated *Aldh2*^−/−^*Fancd2*^−/−^*Trp53*^−/−^ triple knockout mice and performed the clonal HSC mutation burden analysis described above. Surprisingly, while additional knock out of *Trp53* partially rescued the engraftment defect of *Aldh2*^−/−^*Fancd2*^−/−^ HSCs, there was no appreciable increase in the mutation burden of the engrafting clones. These data seem to confirm that acetaldehyde-induced p53 activation does compromise HSC function but suggests that error-prone repair, and the resulting mutations that it generates, are not primary drivers of HSC attrition.

Taken together, this study is the first to evaluate genome-wide clonal mutation spectra for HSCs and, in doing so, has highlighted dietary and endogenous aldehydes as genotoxic agents that can induce mutations in HSCs in vivo. This provides a new molecular basis for understanding at least one mechanism via which excess alcohol consumption may have a broad negative impact on human health. While this work highlights the effectiveness of the partially redundant detoxification system that has evolved to deal with these potent mutagens, it also has important implications for the large number of individuals who carry the dominant negative polymorphism in the *ALDH2* gene.
